# Anterior approach combined with infrahepatic inferior vena cava clamping right hepatic resection for large hepatocellular carcinoma: A prospective randomized controlled trial

**DOI:** 10.1097/MD.0000000000004159

**Published:** 2016-07-08

**Authors:** Yan-Ming Zhou, Cheng-Jun Sui, Xiao-Feng Zhang, Bin Li, Jia-Mei Yang

**Affiliations:** a Department of Hepatobiliary and Pancreatovascular Surgery, First affiliated Hospital of Xiamen University; b Department of Special Treatment, Eastern Hepatobiliary Surgery Hospital, Second Military Medical University, Shanghai, China.

**Keywords:** anterior approach, hepatic resection, hepatocellular carcinoma, infrahepatic inferior vena cava clamping

## Abstract

**Background:**

The anterior approach (AA) technique has been reported to provide better operative and survival outcomes compared with the conventional approach for large right hepatocellular carcinoma (HCC) resection. However, this technique runs the risk of massive retrograde bleeding from the right hepatic vein or middle hepatic vein at the deeper plane of parenchymal transection. This study was designed to evaluate the efficacy of AA combined with infrahepatic inferior vena cava (IVC) clamping on the perioperative outcomes in patients undergoing right hepatic resection for large HCC in randomized clinical trial settings.

**Methods:**

A total of 101 patients undergoing right hepatic resection for large HCC were randomized to receive AA combined with infrahepatic IVC clamping (group A, n = 50), or AA alone (group B, n = 51).

**Results:**

The total blood loss (423 ± 154 vs 757 ± 338 mL; *P* = 0.001), blood loss during liver transection (272 ± 96 vs 563 ± 144 mL; *P* = 0.001), and intraoperative blood transfusion requirements (12.0% vs 29.4%; *P* = 0.031) were significantly less in group A patients compared with group B patients. There was no IVC clamping-associated morbidity in group A.

**Conclusion:**

AA combined with infrahepatic IVC clamping for large right HCC resection is a safe, feasible, and effective technique in reducing intraoperative blood loss.

## 1 Introduction

In conventional right hepatectomy for hepatocellular carcinoma (HCC), complete mobilization of the right liver is performed prior to parenchymal transection. The potential disadvantages of this approach are excessive bleeding from the right liver attachments, iatrogenic tumor rupture, left hepatic lobe ischemia from rotation of the hepatoduodenal ligament, and hematogenous tumor cell dissemination. To avoid these problems, the anterior approach (AA), in which liver mobilization is performed at the end of parenchymal transection, is recommended.^[1]^ Several retrospective studies^[2–4]^ and a randomized clinical trial (RCT)^[5]^ have shown that the AA technique offers better operative and survival outcomes as compared with the conventional approach for patients with large right liver HCC. However, it also runs the risk of massive retrograde bleeding from the right hepatic vein or middle hepatic vein at the deeper plane of parenchymal transection.^[5]^ It is evident that bleeding from the hepatic veins is closely associated with the central venous pressure (CVP).^[6]^ Several researchers have shown that infrahepatic inferior vena cava (IVC) clamping is a simple and effective way to reduce CVP and is associated with significantly less blood loss during hepatectomy.^[7–10]^ This prospective RCT was designed to verify whether AA combined with infrahepatic IVC clamping could decrease intraoperative blood loss in right hepatectomy of large HCC as compared with AA alone.

## 2 Materials and methods

### 2.1 Study design

This prospective RCT was conducted in 2 university centers: the Eastern Hepatobiliary Surgery Hospital of the Second Military Medical University (Shanghai, China) and the First affiliated Hospital of Xiamen University (Xiamen, China). The study protocol was approved by the ethical committees of the 2 centers, and informed consent was obtained from each patient prior to participation. The study was registered at Clinical-Trials.gov (NCT01608386).

### 2.2 Participants

Patients with HCC ≥5 cm in diameter on preoperative imaging who were scheduled for elective and potentially curative major right hepatic resection were eligible for inclusion. Major resection was defined as resection of 3 or more hepatic segments according to the Couinaud nomenclature. Exclusion criteria were age less than 18 years, the presence of extrahepatic metastasis, Child–Pugh grade B or C, emergency surgery, laparoscopic liver resection, chronic obstructive pulmonary disease stage IV, heart failure defined as New York Heart Association class IV, renal insufficiency defined as serum creatinine >2 mg/dL or requiring dialysis, and non-HCC. Portal hypertension was defined as the presence of either oesophageal or gastric varices detectable at endoscopy or thrombocytopaenia (platelet count <100 × 10^9^/L) associated with splenomegaly.

### 2.3 Randomization and masking

Patients were randomized before surgery by an investigator who was not involved in the treatment using a random number table to either group A, where patients received AA combined with infrahepatic IVC clamping, or group B, where patients received AA alone for right hepatectomy.

### 2.4 Study endpoints

The primary endpoint was intraoperative blood loss, which was measured from skin incision until skin closure and estimated by the amount of blood collected in the suction containers including blood squeezed from sponges and gauzes after subtraction of the irrigation volume. The secondary endpoints were blood transfusion requirement, operation time, morbidity, mortality, and the length of postoperative hospital stay. Postoperative adverse events were graded according to the Clavien–Dindo classification.^[11]^ The results were collected and discussed by 2 surgeons who were unaware of the patient treatment groups. Any disagreement was resolved by discussion with a third surgeon.

### 2.5 Surgical technique

An indwelling right subclavian vein catheter and a radial artery catheter were used for hemodynamic monitoring. Surgery was performed via a right subcostal incision. After complete abdominal exploration, intraoperative ultrasonography was used to assess the extent of disease and its relationship with the vascular structures and mark the demarcation line of parenchymal transection. Hepatic hilus dissection was carried out to isolate and divide the right hepatic artery and portal vein. If adequate control of hemorrhage was not achieved by hemihepatic vascular occlusion, the Pringle maneuver was used to control the inflow system. For patients in group A, the infrahepatic IVC was dissected and taped with a vessel loop above the renal veins. In both groups, liver hanging maneuver (LHM) technique was not used. Hepatic parenchymal transection was performed from the anterior liver surface posteriorly toward the IVC along the demarcation line by a clamp crushing method using Kelly forceps without previous mobilization of the right liver. All the small vessels were then individually ligated and divided, and the right hepatic vein was isolated and divided intraparenchymally. When the right hemiliver was completely mobilized from the IVC, the right coronary and triangular ligaments were divided to allow for specimen removal. In group A, complete infrahepatic IVC clamping was performed throughout liver resection including the interval of declamping of the hepatic pedicle.

### 2.6 Power calculation

The sample size was based on the primary outcome: the intraoperative blood loss. A previous RCT showed that the median intraoperative blood loss of the patients who underwent AA major right hepatectomy was 800 mL.^[5]^ To detect a reduction of 150 mL, using a significance level of 0.05 and a power of (1 − β) = 80%, 48 patients were needed in each group. With an estimated dropout rate 5%, the total sample size therefore accounted for 100 patients.

### 2.7 Statistical analysis

Data are presented as patient numbers (percentages) or as the mean ± standard deviation. Categorical variables were compared by using the Chi-square test. Continuous variables were analyzed with the Student *t* test. All statistical analyses were performed using SPSS for Windows (version 11.0; SPSS Institute, Chicago, IL). *P* < 0.05 was considered statistically significant.

## 3 Results

### 3.1 Study sample and patients’ characteristics

**Figure 1:**
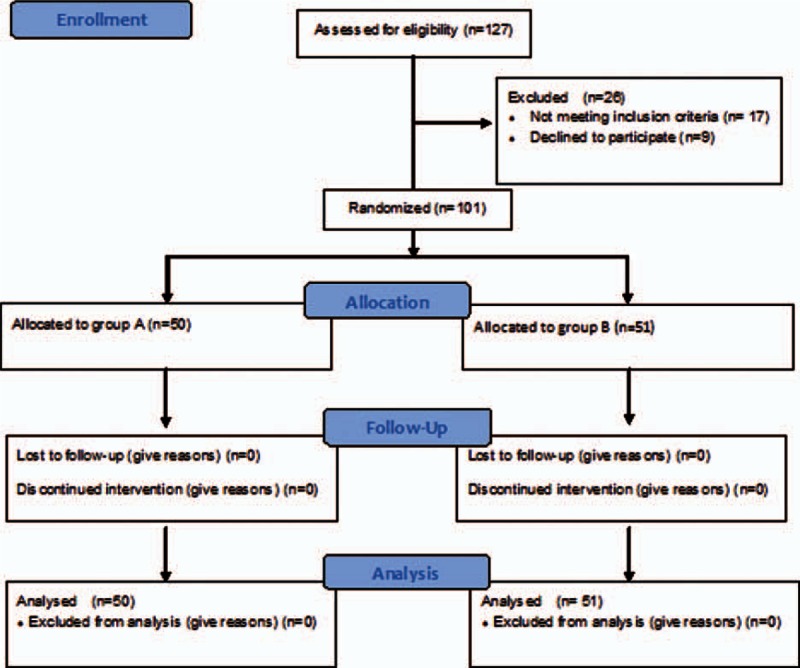
Study flow diagram.

Between June 2012 and March 2015, 127 consecutive patients with large right HCC were evaluated for eligibility, of whom 101 patients were finally included in this study and randomized to group A and B (Fig. 1). The baseline patient characteristics were comparable in both groups (Table 1).

**Table 1:**
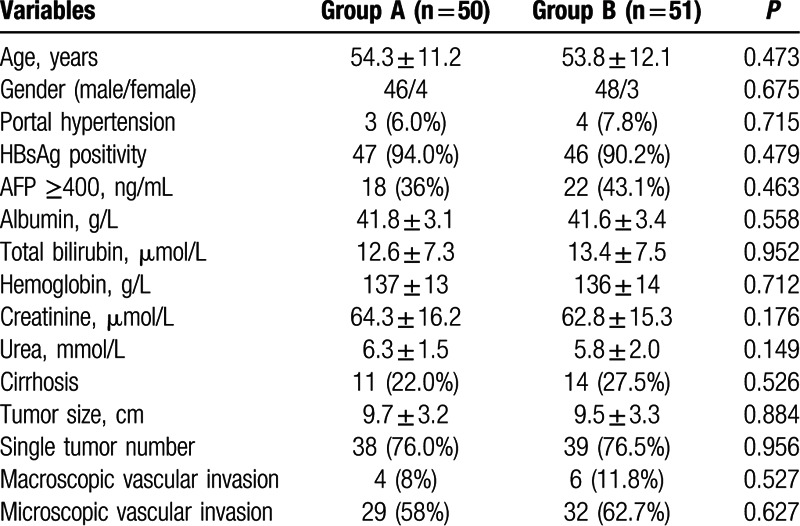
Comparison of patients background characteristics.

### 3.2 Perioperative outcome parameters

Perioperative data of the 2 groups are presented in Table 2. The 2 groups had the similar type of hepatectomy, operative time, and inflow occlusion techniques. However, both the total blood loss (423 ± 154 vs 757 ± 338 mL, *P* = 0.001) and blood loss during liver transection (272 ± 96 vs 563 ± 144 mL, *P* = 0.001) were significantly less in group A patients compared with group B patients. Consequently, group A patients had fewer intraoperative blood transfusion requirements (12.0% vs 29.4%; *P* = 0.031).

**Table 2:**
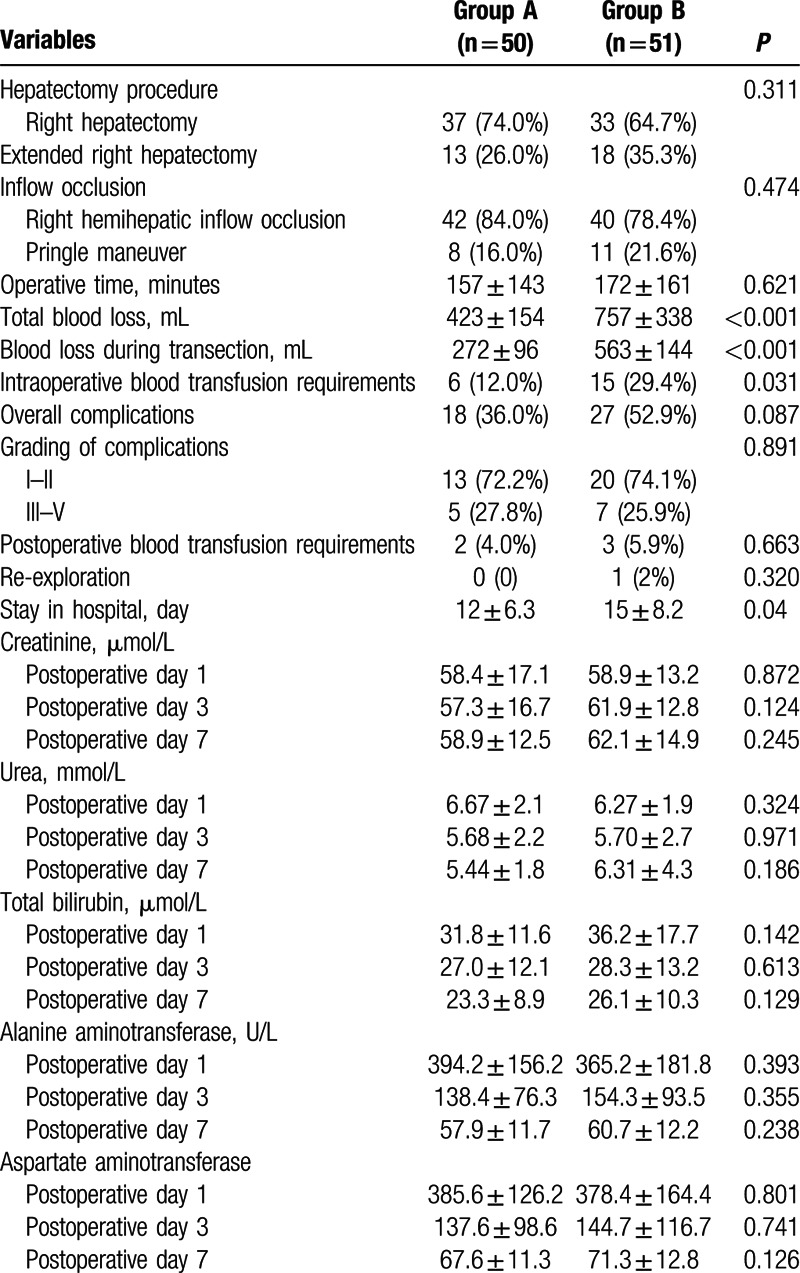
Comparison of intraoperative and postoperative outcomes.

There was a trend toward lower morbidity rate in group A (36.0% vs 52.9%; *P* = 0.087). No significant differences were found between the 2 procedures in terms of the severity of postoperative complications. Postoperative hemorrhage occurred in 2 (4.0%) patients in group A and 3 (5.9%) patients in group B. Three patients presented with intraabdominal hemorrhage and 2 patients presented with upper gastrointestinal tract hemorrhage. All 5 patients received blood transfusion. Four patients were managed nonoperatively and 1 patient in group B underwent reexploration with surgical control of hemorrhage from the cut surface of the liver on the 2nd postoperative day. Patients in group A had shorter hospital stay compared with those in group B (12 ± 6.3 [days] vs 15 ± 8.2 [days]; *P* = 0.04). There was no IVC clamping-associated morbidity including air embolism. No operative death occurred in both groups.

Postoperative renal function was preserved in all patients in the 2 groups. The levels of serum urea and creatinine on postoperative day 1, 3, and 7 were comparable between the 2 patient populations. Similarly, the analysis of the laboratory test of hepatic function demonstrated similar results in both groups.

All patients in group A well tolerated IVC clamping during the mean 54 ± 33 minutes clamping, during which CVP reduced from 8.51 ± 2.56 cm H_2_O to 2.59 ± 1.92 cm H_2_O (*P* < 0.001). IVC clamping did not produce significant impact on the intraoperative heart rate and mean arterial pressure (Table 3).

**Table 3:**
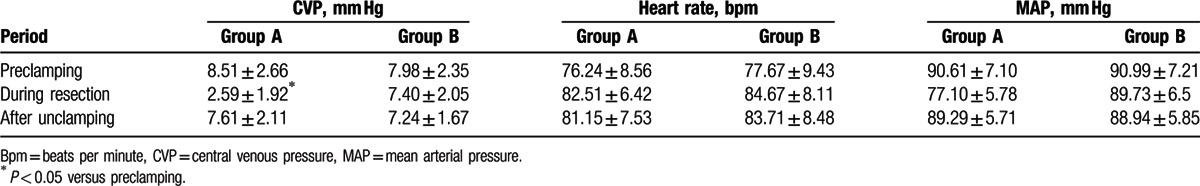
Comparison of intraoperative and postoperative outcomes.

## 4 Discussion

This study was restricted to patients with large HCC (≥5 cm in diameter), a group usually required major hepatectomy.^[12]^ The benefit of AA is evident in that it reduces intraoperative blood loss and transfusion requirements in major right hepatic resection for large HCC.^[5]^ Furthermore, as a “no-touch” technique, AA minimizes the risk of HCC cell dissemination because it avoids tumor mobilization, thus improving the long-term survival outcome of the patient. However, it runs the risk of massive retrograde bleeding from the right hepatic vein or middle hepatic vein at the deeper plane of parenchymal transection, which is often uncontrollable and life-threatening. For this reason, Belghiti et al^[13]^ designed an LHM using a tape inserted between the anterior surface of the vena cava and the liver and combined it with AA in 2001. Although the beneficial effects of this technique have been demonstrated in the literature, including better control of bleeding, protection of the IVC, good exposure during deeper parenchymal dissection, rapid transection, and guidelines for the direction of transection,^[14]^ many surgeons still hesitate to apply this maneuver, for it is technically demanding. In addition, there is still an inherent risk of bleeding from an injured short hepatic vein during retrohepatic blind dissection.^[15]^ Such bleeding is sometimes difficult to control, especially in patients with liver cirrhosis and portal hypertension.^[6]^

Once the inflow blood system of the liver is controlled, the risk of bleeding arises primarily from the hepatic veins during parenchymal dissection. It is generally believed that hepatectomy carried out under low CVP (less than 5 cm H_2_O) will decrease bleeding from the hepatic venous system because the hepatic sinusoidal pressure is directly related to CVP.^[6]^ Conventionally, CVP is reduced by anesthesiological methods such as fluid restriction and additional administration of diuretics, nitro compounds, and opioids if necessary.^[16,17]^ However, restrictive fluid management may compromise hemodynamic stability and associate with intra- and postoperative renal and heart disturbance.

Infrahepatic IVC clamping is a viable option for lowering CVP without the need of systemic fluid restriction. Otsubo et al^[7]^ reported the result of their retrospective study in 103 patients who underwent right or left hemihepatectomy, showing that infrahepatic IVC clamping is very effective in reducing blood loss. An RCT including 128 patients performed by Rahbari et al^[9]^ demonstrated that infrahepatic IVC clamping significantly decreased bleeding and hemodynamic instability events during hepatectomy as compared with anesthesiological methods of CVP reduction.

The present prospective RCT showed clearly that AA combined with infrahepatic IVC clamping reduced intraoperative blood loss significantly as compared with AA alone in patients with large right HCC, especially during parenchymal transection. As a result, fewer patients in group A required blood transfusion. AA for large HCC without prior mobilization of the liver is associated with a difficult to control hepatic venous bleeding. CVP reduction by infrahepatic IVC clamping may circumvent this difficulty. It creates a bloodless field and facilitates identification and control of the intrahepatic structures, thus ensuring an accurate parenchymal transection.

Unlike LHM, dissection and placement of a tape around the infrahepatic IVC are technically safe and simple. Consistent with previous reports,^[7–9]^ our study also showed that IVC clamping was well tolerated by the patients. Even when the infrahepatic IVC is occluded, blood draining from the subphrenic vein, adrenal veins, and lumbar veins may also contribute to the maintenance of hemodynamics. Knowing that low CVP may expose the patients to a high risk of air embolism during liver transection, placing patients in a 15° Trendelenburg position may be a useful maneuver for eliminating this danger. It should also be noted that as the IVC is encircled at a site proximal to the bilateral renal veins, there exists the risk of renal function impairment due to venous blood stagnation. However, there was no significant difference in postoperative serum urea and creatinine values between the 2 groups, suggesting that infrahepatic IVC clamping has little impact on renal function.

## 5 Limitations

The present study has limitations. HCC develops often in cirrhotic liver, which correlates with increased risk of large amounts of blood loss and blood transfusion during hepatectomy.^[18]^ The rate of liver cirrhosis of 25% in this series was low. This can be explained by the fact that our series include only patients selected for major right hepatic resection. Most cirrhotic patients with large tumor were scheduled for limited resection or treated by nonsurgical modality because of preexisting liver damage and therefore were not enrolled in this trial. Similarly, the number of patients with cirrhosis-related portal hypertension was also low in this series. Thus, no subgroup analysis based on these variables could be performed. With limited follow-up, we could not identify a difference in long-term survival between the 2 procedures.

## 6 Conclusions

The evidence from this RCT suggests that AA combined with infrahepatic IVC clamping for large HCC is a safe, feasible, and effective technique for reducing intraoperative blood loss, though larger multicenter trials are needed to validate the conclusion of the present study.

## Acknowledgements

The authors thank Dr Yanfang Zhao (Department of Health Statistics, Second Military Medical University, Shanghai, China) for her critical revision of the statistical analysis section.
